# P67-phox (NCF2) Lacking Exons 11 and 12 Is Functionally Active and Leads to an Extremely Late Diagnosis of Chronic Granulomatous Disease (CGD)

**DOI:** 10.1371/journal.pone.0034296

**Published:** 2012-04-13

**Authors:** Joachim Roesler, Florian Segerer, Henner Morbach, Stefan Kleinert, Sebastian Thieme, Angela Rösen-Wolff, Johannes G. Liese

**Affiliations:** 1 Department of Pediatrics, University Hospital Carl Gustav Carus, Dresden, Germany; 2 Department of Pediatrics, University Hospital of Wuerzburg, Würzburg, Germany; 3 Rheumatology/Clinical Immunology, University Hospital of Wuerzburg, Würzburg, Germany; University of Regensburg, Germany

## Abstract

Two brothers in their fifties presented with a medical history of suspected fungal allergy, allergic bronchopulmonary aspergillosis, alveolitis, and invasive aspergillosis and pulmonary fistula, respectively. Eventually, after a delay of 50 years, chronic granulomatous disease (CGD) was diagnosed in the index patient. We found a new splice mutation in the *NCF2* (p67-phox) gene, c.1000+2T→G, that led to several splice products one of which lacked exons 11 and 12. This deletion was in frame and allowed for remarkable residual NADPH oxidase activity as determined by transduction experiments using a retroviral vector. We conclude that p67-phox which lacks the 34 amino acids encoded by the two exons can still exert considerable functional activity. This activity can partially explain the long-term survival of the patients without adequate diagnosis and treatment, but could not prevent progressing lung damage.

## Introduction

Chronic granulomatous disease (CGD) is caused by inherited defects in the NADPH oxidase multienzyme complex. This rare disorder is associated with life-threatening opportunistic infections and dysregulated inflammation, often accompanied by granuloma formation even in the absence of detectable infections [1]–[6]. In most cases, CGD manifests itself before the third year of life, but a delayed diagnosis, especially in patients with residual NADPH oxidase activity, is quite common [3], [5]. Indeed, more and more cases emerge with manifestations in adulthood. In such cases, the diagnosis is often delayed for years or even decades [6]–[10] preventing adequate treatment.

In addition to infections, older CGD patients frequently suffer from various autoinflammatory symptoms. They need regular medical checkups [11], prophylactic and interventional antimicrobial and/or immunosuppressive treatment [6], [12], [13], and their disorder may be corrected by hematopoietic stem cell transplantation [1], [14]. Gene therapy may be a future therapeutic option [15].

The phagocyte NADPH oxidase is needed for appropriate microbial killing and regulation of inflammation. CGD is caused by mutations affecting the expression or function of one out of four components of this enzyme complex [3]. These components are gp91-phox (also referred to as NOX2), p22-phox, p47-phox, and p67-phox, (MIM#s 608515, 233710; -phox, phagocyte oxidase). Rac2 [16], p40-phox, and severe G6PD deficiency also cause CGD-like diseases, but differ from the classical form. In about two-thirds of all CGD cases, mutations are found in the X-chromosomal *CYBB* gene encoding gp91-phox/NOX2. The genetic aberrations are family-specific and comprise a wide range of mutation types [17]. Mutations are also family-specific in autosomal-recessive p22-phox [18] and in p67-phox [19] deficiencies, which are much rarer than the X-linked form (each 5% of all CGD cases). In contrast, autosomal recessive p47-phox deficiency (25% of all CGD cases [19], [20]) is mostly due to recombination events between the *NCF1* gene and one out of two highly homologous pseudogenes, thus leading to the same GT deletion at the beginning of exon 2 in 80–90% of all p47-phox–deficient CGD patients.

In healthy individuals, the p67-phox protein combines with other components of the NADPH oxidase to form the fully-functional reactive oxygen species (ROS)-producing enzyme complex [21], [22]. The SH3 domain close to the C-terminal end of p67-phox interacts with the proline-rich region (PRR) of p47-phox, the PB1 domain links p67-phox to p40-phox, and the tetratricopeptide repeat (TPR) region of p67-phox domain binds Rac-GTP [21], [23].

Here we describe a new splice mutation in *NCF2* (p67-phox) leading to residual NADPH oxidase activity, thereby contributing to an extremely late diagnosis of CGD in adulthood.

## Results

### Case reports

At age 8 years, the index patient was first hospitalized for six months with a fungal pneumonia after threshing of mouldy grain. Thirty years later, he had another fungal pneumonia caused by non-specified *Aspergillus*. Thereafter, he suffered from recurrent episodes of dyspnea mostly after exposure to moulding organic material (**Table 1**). These episodes were interpreted as fungal allergy, allergic bronchopulmonary aspergillosis or hypersensitivity pneumonitis and treated with steroids and antimycotic drugs. At age 48 years a persisting pulmonary fistula and local infiltration with *Aspergillus fumigatus* prompted lobectomy of the lower left lung lobe. Between age 54 and 56 years two invasive pulmonary *Aspergillus* infections of the right and left upper lobe and a fistula of the left upper lobe were treated by dissection of the affected lung parts. Prolonged immunosuppression by steroids was thought to be the reason for these complications. However, after discontinuation the patient experienced a rapid deterioration of his pulmonary function requiring continuous oxygen supplementation and causing cor pulmonale.

**Table 1:** Overview over medical histories.

	*Medical history of patient 1; 58 years old at diagnosis of CGD*	
Age (years)	Symptoms	Therapy
Before 8 *	Eczema, recurrent tonsillitis	Tonsillectomy
8*	Fungal pneumonia	Antimycotics for 6 months
38 (and later)	Suspected pulmonary aspergillosis	Intermittent treatment with itraconazole and corticosteroids
38	Documentation of chronic inflammatory lung disease	Starting long term treatment with corticosteroids, further intermittent treatments with antimycotics, especially itraconazole
39	Suspected hypersensitivity pneumonitis	
47	Suspected allergic bronchopulmonale aspergillosis	
48	Lung biopsy complicated by fistula formation, *Aspergillus fumigatus* found	Resection of left lower lobe
51–54	Recurrent pulmonary aspergillosis	At age 54: Resection of right upper lobe infiltrated by A*spergillus fumigatus*
56	Aspergillosis of left upper lobe	Resection of left segments 1–3, complicated by fistula formation
56	Unspecified pneumonia	Addition of antibiotics
58	Pulmonary deterioration after discontinuation of corticosteroids: diagnosis of pulmonary hypertension and partial respiratory insufficiency	Home oxygen supply

	*Medical history of patient 2; 53 years old at diagnosis of CGD*	
Age (years)	Symptoms	Therapy
Whole childhood*	Recurrent skin abscesses	
3*	Fungal pneumonia	Antimycotics for 6 months
41–48	Recurrent dyspnea after grain dust exposure (ale brewer), suspected exogen allergic alveolitis	Intermittent treatment with corticosteroids
48	Official recognition of “severe grain dust allergy” as work-related disease	
48	Liver abscess by *Staph. aureus*	Drainage

* The medical histories of childhood are slightly vague because no documents were available, but rely on what the patients and their mother remember.

The 53 year-old brother of the 58 year-old index patient had to give up his profession as a beer brewer due to recurrent episodes of dyspnea after exposure to moulding organic materials. These episodes were thought to be of allergic etiology. Furthermore, a liver abscess caused by *Staph. aureus* was drained. (**Table 1**, bottom).

### Laboratorial findings

**Figure 1:**
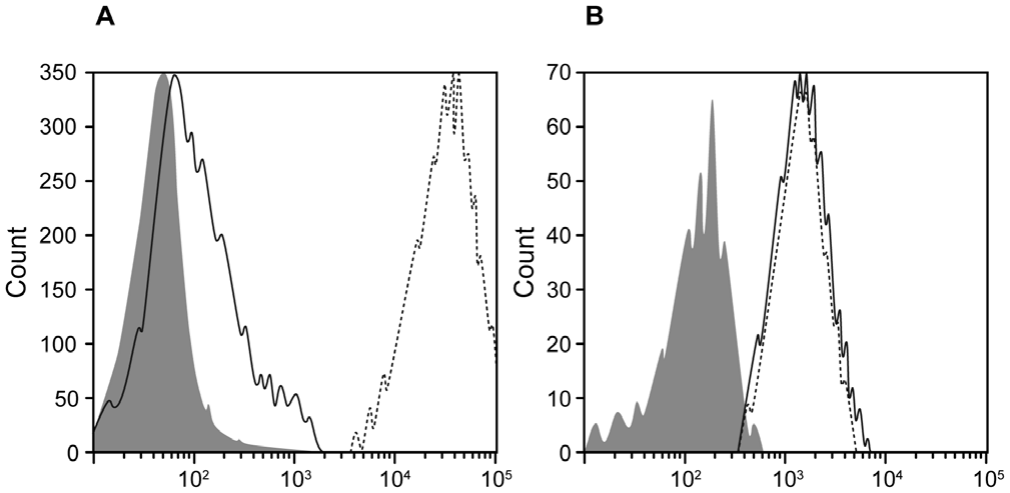
Production of small amounts of ROS (hydrogenperoxide) by neutrophils from the index patient (A, straight line) **and normal expression of cytochrome b558** (consisting of gp91-phox and p22-phox, B). **A**, DHR assay; no stimulation, gray area; activation with PMA, patient's cells, straight line; cells from a healthy control donor, dotted line; the residual NADPH oxidase activity was reproducible in different labs. **B**, cell staining with the mab 7D5; gray area, isotype control; lines as in A. Abscissa, green fluorescence.

To diagnose CGD, reactive oxygen species (ROS) were measured using the DHR assay and lucigenine enhanced chemoluminescence (CL) [24]. Both tests showed small amounts of residual NADPH oxidase activity (**Fig. 1, A** **;** **Table 2**). Neutrophils and monocytes from the index patient expressed cytochrome b558 normally as revealed by staining with the mab 7D5 and flow cytometry (**Fig. 1, B**). In the majority of CGD cases, leukocytes are cytochrome b558 negative when mutations are located in the membrane associated components gp91-phox (*CYBB*) and p22-phox (*CYBA*) of the NADPH oxidase, but always positive when the mutations are located in the cytosolic factors of this enzyme. Consequently, we focussed on these factors and sequenced exon 2 of the *NCF1* (p47-phox) gene to check for the hot spot mutation c.75_76delGT [20], and the *NCF2* gene on the genomic level.

**Table 2:** Residual ROS (superoxide) production by 10^5^ neutrophils.

CL	Indexpatient	Healthy donor
unstimulated	1.5×10**^6^**	2.8×10**^6^**
PMA	2.8×10**^6^**	28.0×10**^6^**
Zymosane A	3.7×10**^6^**	21.0×10**^6^**

CL: lucigenine enhanced chemoluminescence, arbitrary light units in 30 min. PMA: phorbol myristate acetate.

The index patient and his brother were normal at the p47-phox hot spot, but homozygous for a splice mutation (c.1000+2T→G) downstream of exon 11 in *NCF2*. This mutation destroyed the splice consensus sequence. Accordingly, several deranged splice products, but no normally spliced NCF2 cDNA could be detected by separation on an agarose gel (data not shown). cDNA strands of different length were cut out and sequenced. However, many bands consisted of heterodimers of different splice products complicating the analysis. As expected, exon 11 was always skipped and one main splice product lacked exon 11, but was otherwise normal.

**Figure 2:**
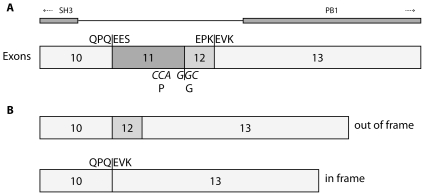
Schematic representation of the normal exon arrangement (A) and two main splice products in the patients (B). Italic letters represent the bp sequence at the exon 11/12 junction, the other capital letters represent the amino acid sequences at exonic junctions. The deletion of exons 11 and 12 does not affect a known functional domain (A, top), but may shorten the distance between the first SH3 domain (possibly binding to gp91-phox) and the PB1 domain (binding to p40-phox).

**Figure 3:**
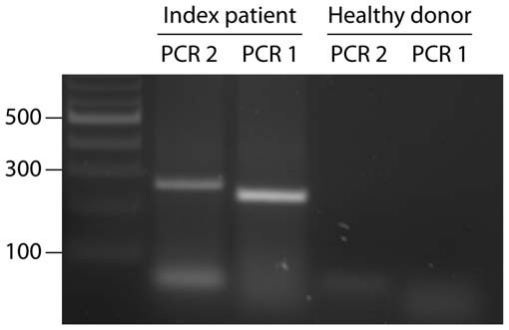
PCR with primers (**Table 1**) overlapping exons 10 and 13 gave amplimers only when applied to patient's cDNA, but not when applied to cDNA from a healthy donor. The correct sequence of the amplimers was confirmed. Ordinate: length in bp.

However, NCF2 mRNA without exon 11 could not account for the residual NADPH oxidase activity because this exon skipping deranged the sequence by setting it out of frame (**Fig. 2, B**). Further search raised suspicion for a splice product lacking two exons, 11 and 12 (p67delex11,12) and thereby 34 triplets. The presence of this splice product was confirmed by two PCR approaches, one using a forward primer located in exon 10 with an overlap into exon 13 and a downstream reverse primer, and a second PCR using a reverse primer in exon 13 with an overlap into exon 10 and an upstream forward primer (PCR 1, 2, **Table 3**, **Fig. 3**). Both PCR approaches gave strong bands when applied to patient cDNA, but gave no amplimer when applied to normal cDNA **Fig. 3**). The splice product p67delex11,12 was the only one found to be in frame (**Fig. 2**).

**Table 3:** Primers overlapping exons 10 and 13 to confirm the presence of p67delex11,12.

PCR	Upstream	Exon 10	Exon 13	Downstream
**1**		AGCAGCAGCCCCAG	GAAGT→	←CAGCATGAAGGATGCCTG
**2**	CTCCACCCAGACCGAAAAC→	←CCAG	GAAGTGAAGCTCAGTGTT	

The forward sequence of the reverse primers is shown.

In order to confirm that p67delex11,12 could indeed lead to a functional NADPH oxidase, we inserted a p67delex11,12-cDNA into a retroviral vector and transduced K562 cells expressing all NADPH oxidase components except p67-phox (model p67-CGD cells). As shown in **Table 4** the NADPH oxidase multi-enzyme-complex that contained p67delex11,12 instead of complete p67-phox could still produce remarkable amounts of ROS (approximately 10% superoxide compared to the wild-type form) after stimulation with phorbol myristate acetate (PMA).

**Table 4:** NADPH oxidase with p67delex11,12 instead of complete p67-phox yields ROS.

AComplete NADPH oxidase			CModel p67-CGD cells+p67-phox		
PMA	**−**	**+**	PMA	**−**	**+**
	**4.4**±1.9	**3590**±1600		**3.4**±0.3	**1950**±670

BModel p67-CGD cells			DModel p67-CGD cells+p67delex11,12		
PMA	**−**	**+**	PMA	**−**	**+**
	**1.6**±0.4	**2.4**±0.8		**2.5**±0.6	**204**±80

A, K562 cells that permanently express all components of the NADPH oxidase. B, same cells, but lacking p67-phox. C, same cells as in B, but transduced with complete p67-phox. D, same cells as in B, but transduced with p67delex11,12. Arbitrary light units ×10**^6^** over 45 min; 5 independent transductions.

IFNγ has been described to influence splicing and nuclear export of normal transcripts in a case of gp91-phox deficient CGD caused by a splice mutation [25]. To analyze if IFNγ could also be effective in our patients, we incubated EBV-transformed B cells from the index patient with 10 or 20 ng/ml IFNγ [25], but found no improvement in the production of ROS (data not shown).

## Discussion

We report two brothers who suffered from symptoms typical for CGD, starting with the first manifestation of their disorder in childhood (**Table 1**). The extreme delay of 50 years between the first symptom and the diagnosis of CGD in the index patient may be explained by the severe inflammatory lung disease, a condition erroneously not considered to be typical for primary immunodeficiency. Unfortunately, physicians other than specialized pediatricians are seldom familiar with CGD, which is a rare disorder (approx. 1 in 200,000 newborns).

The prognosis of CGD patients clearly depends on residual NADPH oxidase activity [26]. However, there are other environmental and genetic effects that also influence the outcome [27]. The ROS production of neutrophils from our patients was very low. Nevertheless, it has most probably helped to slow down the progression of CGD complications.

The patients were homozygous for the new disease-causing splice mutation (c.1000+2T→G) and their parents therefore most probably consanguineous. The mutation could not easily explain the residual NADPH oxidase activity. It predicts skipping of exon 11 that was found indeed, but sets the downstream mRNA out of frame. Splice mutations often generate several aberrant splice products for example by indirectly activating cryptic splice sites. However, such a splice site could not be found, neither *in silico* nor by sequencing.

Skipping of two (or more) exons does also occur when splicing is impaired. We detected a splice product, p67delex11,12, that was absent from cDNA of healthy donors. This splice product was a candidate mRNA that could possibly account for the residual NADPH oxidase activity because it remained in frame and the deletion did not affect a known functional domain [21], [22], **Fig. 2**. A transduction experiment using a retroviral vector and model p67-CGD cells could indeed substantiate this assumption. p67delex11,12 supports considerable ROS production (approximately 10% of normal, **Table 4**). This finding contrasts to skipping of exon 5 that leaves also the p67-phox mRNA in frame, but leads to a non-functional protein variant that is rapidly degraded [28].

Interestingly, the exons 11 and 12 of the *NCF2* gene are not highly conserved in the evolution of vertebrates that all have orthologues of p67-phox [29]. The genome of the lizard *Anolis carolinensis* lacks exons homologous to the human exons 11 and 12, but has exonic sequences homologous to all other human *NCF2* exons that are translated (according to ENSEMBL Genomic alignments, http://www.ensembl.org). *Anolis carolinensis* could be representative for other reptiles, but this has not yet been checked. Frogs and fish have also no homologies to exons 11 and 12, but lack some additional homologies to human *NCF2* exons.

The low residual NADPH oxidase activity in the patients in spite of the considerable functional potential of p67delex11,12 can be easily explained by the fact that only a small portion of aberrant mRNA consisted of p67delex11,12. In an agarose gel, the respective cDNA did not form a clear band, but formed heterodimers with other splice products and was presumed by analyzing sequence-overlays. Only the specific PCRs (**Fig. 3**) revealed clearly the existence of p67delex11,12.

IFNγ has been described to improve fidelity of splicing and nuclear export of normal transcripts in a case of gp91-phox deficient CGD caused by a splice mutation [25]. If IFNγ increased the inclusion of exon 12 into p67-phox mRNA in our case, this would of course not enhance the production of ROS. In contrast, an increased nuclear export of p67delex11,12 could lead to such an improvement. However, no increase in ROS generation could be induced by IFNγ in EBV-transformed B cells from the index patient.

From our findings we draw i) a biochemical and ii) a clinical conclusion. i) Even though exons 11 and 12 of p67-phox are necessary for an optimal function of the human NADPH oxidase, the p67-phox-protein can work without the amino acid stretch encoded by these exons. ii) Our article further contributes to the notion that CGD should be considered at any age of patients presenting with symptoms that fit this disorder such as severe bacterial or fungal infections or inflammatory symptoms [6]–[10]. Failure to diagnose the disease can lead to progression of complications and organ failure whereas an early diagnosis can be prompted by adequate prophylaxis, regular check ups, and in many cases curative hematopoietic stem cell transplantation (HSCT). The standard prophylaxis comprises co-trimoxazole and itraconazole. IFNγ s.c. may be added irrespective of its ex vivo impact on cells from patients [30]. Reduced intensity conditioning HSCT is always indicated if standard prophylactic therapy has an insufficient effect, but may be a favourable treatment after the first severe infection or, in certain cases, even before the first severe manifestation of CGD has occurred [31].

## Materials and Methods

The patients gave written consent to blood drawing and all diagnostic testing, including genetic analysis. Our study has been approved by the following ethics committee: Ethik-Kommission bei der Medizinischen Fakultät der Universität Würzburg.

### Chemiluminescence assays for NADPH Oxidase Activity

Superoxide (O_2_ **^−^**) production in transduced 5×10**^5^** K562 cells and in EBV-transformed B cells was measured by chemiluminescence over 45 min after stimulation with 1 mg/mL phorbol-12-myristate-13-acetate (PMA) in a luminol-based chemiluminescence cocktail (Diogenes; National Diagnostics, Atlanta, GA) in a Mithras LB 940 microplate reader (Berthold Technology, Bad Wildbad, Germany). For patients' neutrophils lucigenine was used instead of the Diogenes reagent.

### Determination of Flavocytochrome b558 Expression and H_2_O_2_ Production at the Single-Cell Level

The dihydrorhodamine-1,2,3 flow cytometry assay (DHR assay) for NADPH–oxidase-dependent production of ROS in neutrophils, as indicated by H_2_O_2_ production, was used as described [24]. Staining with the monoclonal antibody (moAb) 7D5, which specifically binds to an extracellular epitope of gp91-phox, was performed according to standard techniques. Samples were analyzed by flow cytometry (FACS Calibur; BD Biosciences, San Jose, CA).

### K562 Cell Line, Culture Conditions, and Incubation of EBV transformed B Cells with IFNγ

We used a human K562 cell model of p67-phox–deficient CGD (model p67-CGD cells) that was engineered to contain p47-phox and gp91-phox (and that naturally expresses p22phox mRNA). Only when K562-67def-CGD cells are transduced to also produce p67-phox do these cells become capable of generating superoxide in response to phorbol 12-myristate 13-acetate (PMA) stimulation [32]. The cells were cultured in RPMI-1640 medium supplemented with 10% (v/v) fetal bovine serum (FBS), 2 mM glutamine, 100 U/mL penicillin, and 0.1 mg/mL streptomycin. EBV-transformed B cells from the index patient and a healthy control donor were incubated with IFNγ (10 or 20 ng/ml) for seven days as described [25].

### PCR and Sequencing

Genomic DNA was isolated with the QIAamp DNA Blood kit (Qiagen, Hilden, Germany). PCR reactions were performed with AmpliTaq Gold DNA Polymerase (Applied Biosystems, Warrington, UK). DNA analysis was performed with the ABI BigDye terminator-cycle-sequencing kit (Perkin Elmer, Weiterstadt, Germany) and an ABI 3130XL automatic sequencer (Applied Biosystems, Carlsbad, CA, USA). Aberrant sequences were confirmed on both DNA strands and in a second DNA sample to avoid PCR artifacts. The ENSEMBL NCF2 (ENSG00000116701) sequence was used.

Special primers were used for the verification of a splice product lacking exons 11 and 12 (**Table 3**). A forward primer was located in exon 10 with a 5 bp overlap into exon 13 and was combined with a downstream reverse primer, and a reverse primer in exon 13 had a 4 bp overlap into exon 10 and was combined with an upstream forward primer.

### Retroviral Vectors and Vector Production

Full-length p67-phox-cDNA was ligated into NdeI/XhoI-opened pET15b (Novagen; Merck Chemicals, Darmstadt, Germany), to obtain pET15b-p67. To remove exons 11 and 12, we applied overlap extension PCR and ligated the final PCR product into AvrII/NcoI-opened pET15b-p67 to obtain pET.p67delex11,12. Full-length p67-phox and p67delex11,12 were PCR amplified and cloned into a gammaretroviral transfer vector [33] (MFGS). Virus-particle-containing medium was from HEK 293T cells cotransfected in 10-cm dishes with 10 mg of transfer vector (pM.p67.iG or pM. p67delex11,12.iG), 2 mg pMD.G (vesicular stomatitus virus glycoprotein envelope plasmid), and 6.5 mg of pHIT60 (gagpol-packaging plasmid) in the presence of 6.75 mg/mL polyethyleneimine. K562 cells were transduced with virus-containing media in the presence of protamine sulfate (5 mg/mL) and by using spinocculation (1,200 g, 30 min).
